# Generation of Active Neurons from Mouse Embryonic Stem Cells Using Retinoic Acid and Purmorphamine

**DOI:** 10.3390/ijms26178372

**Published:** 2025-08-28

**Authors:** Ruby Vajaria, DeAsia Davis, Francesco Tamagnini, Duncan G. G. McMillan, Nandini Vasudevan, Evangelos Delivopoulos

**Affiliations:** 1 School of Biological Sciences, University of Reading, Reading RG6 6UB, UKdeasiaadavis@gmail.com (D.D.); d.g.g.mcmillan@reading.ac.uk (D.G.G.M.); n.vasudevan@reading.ac.uk (N.V.); 2 School of Pharmacy, University of Reading, Reading RG6 6UR, UK; f.tamagnini@reading.ac.uk

**Keywords:** embryonic stem cells (ESCs), neural differentiation, Leukemia Inhibitory Factor (LIF), retinoic acid, cell culture, neuron

## Abstract

Multiple differentiation protocols have emerged in recent years, producing neurons with diverse morphologies, gene and protein expression profiles, and functionality. Many of these differentiation techniques require months of culture and the use of expensive growth factors. Most importantly, the derived neurons usually do not exhibit any electrical activity. This limits the value of the protocol as a tool for engineering and investigating neural networks. Here, we describe an efficacious method for differentiating mouse embryonic stem cells into functional neurons. CGR8 cells were neurally induced via the simultaneous application of retinoic acid and purmorphamine. The derived cells expressed neuronal (TUJ1 and NeuN) and synaptic (GAD2, PSD-95, Synaptophysin, and VGLUT1) markers. During whole-cell recordings, neurons exhibited inward and outward currents, likely caused by fast-inactivating voltage-gated potassium channels. Upon current injection, miniature action potentials were also recorded. The efficient generation of diverse subtypes of functional neurons can be a useful tool in fundamental investigations of neural network activity and translational studies.

## 1. Introduction

Over the past two decades, numerous techniques have emerged for differentiating stem cells into different types of neurons. Examples of the produced cells include hypothalamic [1], dopaminergic [2], cholinergic [3], cortical glutamatergic or GABAergic [4], and motor neurons [5]. Stem cell differentiation protocols either synchronously or serially apply transcription factors and signaling molecules at fine-tuned concentrations and a precise timeline during culture. This is performed in order to emulate signaling pathways that are present during embryonic development. The end goal is to activate a set of genes that promotes a specified developmental trajectory toward a target neuronal identity [6].

There are three main ways of neuralizing stem cells: via embryoid bodies [7], monolayer cultures [8], or exosomes [9,10]. Amongst others, the following proteins and their respective genes are used to characterize the produced cells as neurons: PAX6, Nestin, MAP2, SOX1/2, OLIG2, ChAT, SYN, and TUJ1 [11]. The formation of excitatory and inhibitory synapses within developing networks is usually identified via the VGLUT, VGAT, and GAD2 markers [12], whereas select studies report on the electrophysiological properties of the produced neurons and their ability to generate action potentials [13]. The maturation stage of the differentiated neurons is also an important factor, as it dictates how suitable they are for transplantation and whether they will establish long-distance projections once transplanted [14].

The CGR8 mouse embryonic stem cell (mESC) line was derived from the inner cell mass of a 3.5 day male pre-implantation mouse embryo (*Mus musculus*, strain 129). This is an established mESC line that has been used to generate cardiomyocytes [15,16], astrocytes [17], leucocytes [18], adipocytes [19], and neural progenitors [20], amongst other cell types. CGR8 cells have been characterized in terms of the ChAT [21] and estrogen receptor [22] distribution, adhesion on silicon substrates [23], as well as different cell surface markers (CD45, CD68, CD19, CD11b, etc.) [24]. CGR8 pluripotency is easily preserved with the addition of LIF (Leukemia Inhibitory Factor) in the media, and unlike other cell lines, CGR8 do not require a feeder layer (murine embryonic fibroblast independent) [25]. As a result, CGR8 are increasingly used with different natural [26] and synthetic [27] scaffold materials in tissue engineering [28,29].

Retinoic acid (RA) is essential for neural specification of the dorso-ventral and rostro-caudal axes of the neural tube. RA is metabolized from retinol inside the cytoplasm through the action of retinaldehyde dehydrogenase (Raldh1/2/3) and subsequently binds to retinoic acid receptors (RARs), which in turn form heterodimers with retinoid X receptors (RXRs). Through RXRs, RA induces rapid activation of transcriptional cascades, including the induction of Hox gene clusters. As a result, RA promotes the differentiation of neurons, particularly of a caudal and dorsal identity; hence, an abundance of neural differentiation protocols rely on the inclusion of RA in the medium [30,31,32]. On the other hand, Sonic hedgehog (Shh) is a ventralizing morphogen expressed by the notochord and the floor plate of the neural tube. It is essential for establishing dorso-ventral neural patterning during development via the regulation of Hox proteins in ventral progenitor domains. Shh actions are transduced via the Smoothened (Smo) transmembrane protein, which is regulated by a separate transmembrane receptor called Patched (Ptc) [33]. During embryogenesis, the patterning of MNs is directed by the interaction of RA and Shh, which, respectively, caudalize and ventralize differentiating neural progenitors [34,35].

In this study, we differentiated the mouse embryonic stem cell line CGR8 into cells expressing neuronal markers and exhibiting action potentials (mESns), based on a protocol established by Wichterle and Peljto [32]. CGR8 cells start as pluripotent, as verified by the expression of the Oct4 transcription factor. They are subsequently neurally induced via the traditional embryoid body (EB) formation technique and directed toward a neuronal lineage. The inclusion of retinoic acid and purmorphamine in the differentiation media suppresses the expression of Oct4 and upregulates proneural genes. Further application of B27 and BDNF leads to the efficient conversion of mESCs to neuron-like cells (mESns) within 2 weeks.

## 2. Results

### 2.1. Immunocytochemical Analysis of mESC-Derived Neurons

**Figure 1:**
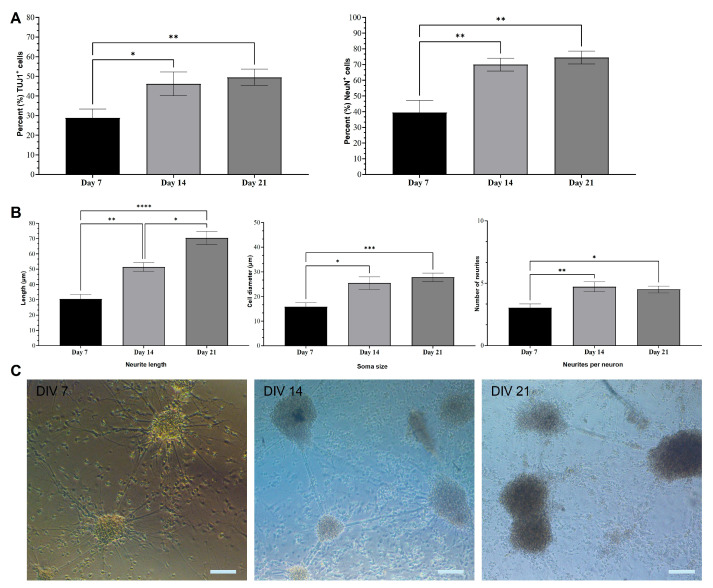
Quantification of neuronal marker expression and morphological characterization of CGR8 cell-derived neurons. (**A**) Percentages of TUJ1^+^ and NeuN^+^ cells at DIV 7, DIV 14, and DIV 21 (**B**) Average neurite length, soma size, and neurites per neuron of TUJ1^+^ neurons at DIV 7, DIV 14, and DIV 21. (All data are expressed as the means ± SEMs from three separate experiments, *n* = 6, * *p* < 0.05, ** *p* < 0.01, and *** *p* < 0.001). (**C**) Brightfield images illustrating developing neuronal networks at DIV 7, DIV 14, and DIV 21. Neurons are organized in interconnected clusters from DIV 7. The density of clusters increases at DIV 14. In DIV 21 networks, more “free” cells are noticeable (not in clusters). Scale bars: 100 μm.

The mESC induction and differentiation protocol generated a heterogeneous population of cells. The formed embryoid bodies were broken down and triturated to release neurally induced cells that were plated onto laminin-coated coverslips and cultured in neuronal medium. Differentiating cells started forming neuronal clusters by the end of the first week in culture (Figure 1C). At the end of the second week, all clusters were interconnected and covered the entire coverslip. A high-magnification brightfield image of DIV 21 neurons (Figure 1C) highlights individual cell bodies and neurites, as well as fascicles connecting growing clusters.

**Figure 2:**
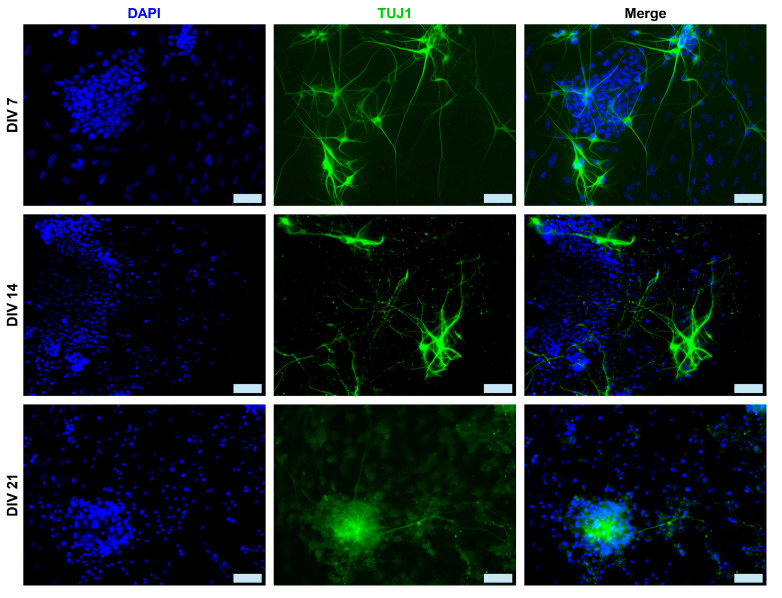
Micrographs (DIV 7–21) of cultured neurons induced from mESCs. Examples of TUJ1^+^ (green) mESC-differentiated neurons at DIV 7, 14 and 21. Stained neurons have a typical apical morphology and extend multiple processes (e.g., DIV 7). Scale bars: 50 μm.

**Figure 3:**
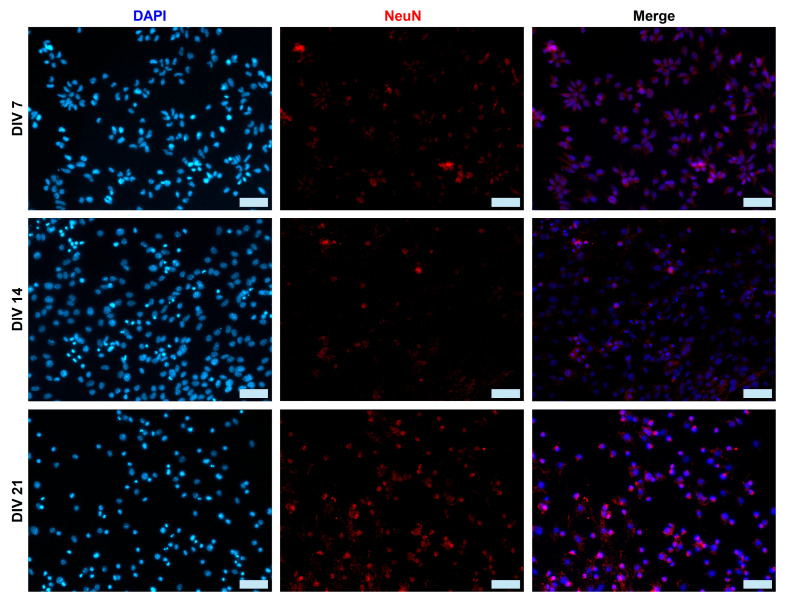
Micrographs (DIV 7–21) of cultured neurons induced from mESC. Examples of NeuN^+^ (red) mESC-differentiated neurons at DIV 7, 14 and 21. At DIV 14 and 21, the presence of both NeuN^+^ and NeuN^−^ cells is evident. Scale bars: 50 μm.

We used the two main neuronal markers β-Tubulin-III (TUJ1 is expressed earlier in neuronal differentiation) and NeuN to characterize the mESC-derived neurons at DIV 7, 14, and 21. Individual TUJ1^+^ neurons and their extended neurites are still visible at DIV 7, whereas by DIV 21, the majority of neurons are clustered (Figure 2). On the other hand, NeuN^+^ neurons appear to be organized in rosettes at DIV 7, evolving into a carpet structure by DIV 21 (Figure 3). We measured the percentage of the total cell population expressing either TUJ1 or NeuN and we saw a marked increase in both markers throughout the 3 weeks of culture. A one-way ANOVA followed by the Kruskal–Wallis test comparing DIV 7, DIV 14, and DIV 21 was conducted. There was a significance increase in the number of TUJ1^+^ cells from DIV 7 (28.8% ± 4.6%, *n* = 6) to DIV 14 (46.2% ± 6.0%, *n* = 6) (*p* = 0.0409) and DIV 21 (49.5% ± 4.1%, *n* = 6) (*p* = 0.0055). There was also a significant increase in the number of NeuN^+^ cells from DIV 7 (39.5% ± 7.7%, *n* = 6) to DIV 14 (70% ± 4.1%, *n* = 6) (*p* = 0.0094) and DIV 21 (74.47% ± 4.1%, *n* = 6) (*p* = 0.0026), as shown in Figure 1A.

**Figure 4:**
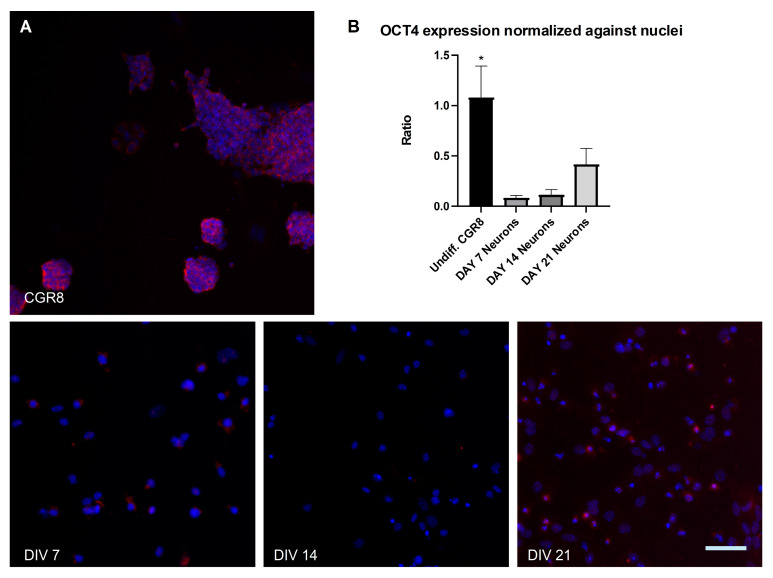
Micrographs of cultured neurons induced from mESCs and undifferentiated CGR8 cells. (**A**) Examples of OCT4^+^ (red) undifferentiated mESCs and OCT4^-^ neurons at DIV 7, 14, and 21. (**B**) Quantification of OCT4 expression reveals a significant decline in the pluripotency marker in differentiated neurons. (All data are expressed as the means ± SEMs from three separate experiments, *n* = 3, * *p* < 0.05). Scale bar: 100 μm.

The pluripotency marker Oct4 was examined throughout the 3 week culture period (Figure 4A). The expression of Oct4 in DIV 7 (0.09 ± 0.02, *n* = 3), DIV 14 (0.12 ± 0.05, *n* = 3) and DIV 21 (0.42 ± 0.16, *n* = 3) differentiated neurons was contrasted with Oct4 expression in undifferentiated CGR8 cells (1.08 ± 0.31, *n* = 3) and a statistically significant decrease was detected (*p* = 0.0139) (Figure 4B).

### 2.2. Morphological Characterization of mESC-Derived Neurons

Neurons derived from CGR8 mESCs were also characterized morphologically by measuring the neurite length, soma size, and number of neurites per TUJ1^+^ neuron at DIV 7, 14, and 21. A one-way ANOVA (Kruskal–Wallis test) comparing different time points revealed a significant increase in neurite length from DIV 7 (30.6 ± 3.1 μm, *n* = 6) to DIV 14 (51.4 ± 2.9 μm, *n* = 6) (*p* = 0.0025) and DIV 21 (70.5 ± 4.4 μm, *n* = 6) (*p* < 0.001), as shown in Figure 1B. Neurite lengths of DIV 14 and 21 neurons were also statistically different (*p* = 0.0423), which highlights the sustained elongation of neurites as neurons mature in culture. Similarly, there was a significant increase in neuronal soma size (Kruskal–Wallis test) from DIV 7 (15.8 ± 1.7 μm, *n* = 6) to DIV 14 (25.4 ± 2.5 μm, *n* = 6) (*p* = 0.019) and DIV 21 (27.8 ± 1.7 μm, *n* = 6) (*p* < 0.001), as shown in Figure 1B. There was no statistically significant difference in soma size between DIV 14 and DIV 21 neurons. Finally, the number of neurites per neuron increased from DIV 7 (3 ± 0.3, *n* = 6) to DIV 14 (4.7 ± 0.4, *n* = 6) (*p* = 0.0041) and DIV 21 (4.5 ± 0.3, *n* = 6) (*p* = 0.0219). There was no statistically significant increase in the number of neurites per neuron between DIV 14 and DIV 21 neurons (Figure 1B).

### 2.3. Expression of Synaptic Markers in CGR8 Cell-Derived Neurons

**Figure 5:**
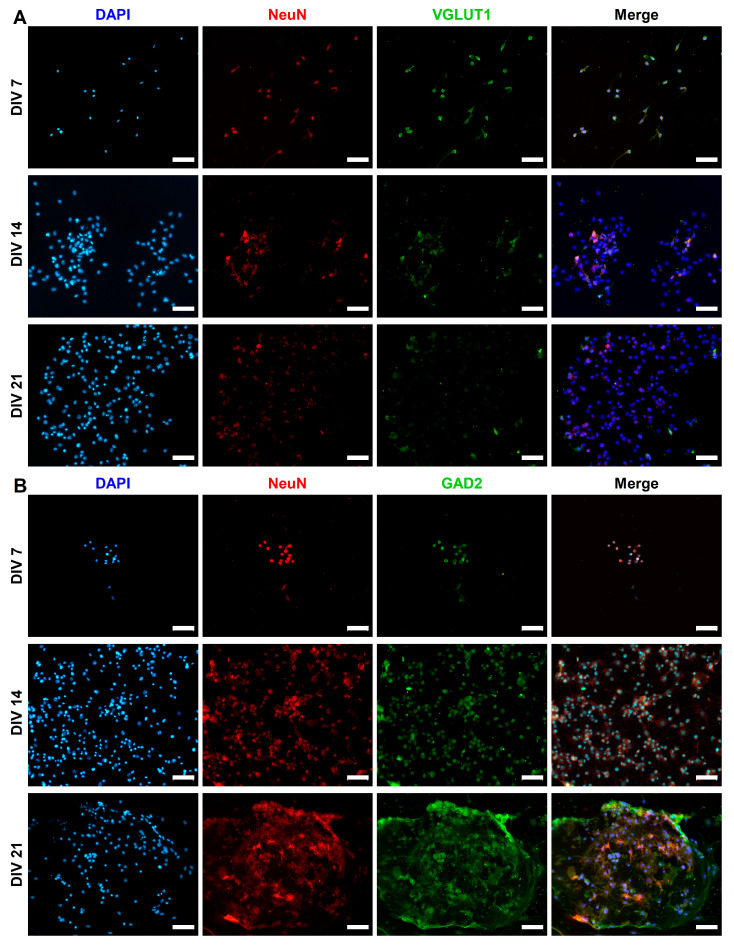
Examples of (**A**) VGLUT1 and (**B**) GAD2 expression in NeuN^+^ (red) mESC-differentiated neurons at DIV 7, 14, and 21. The majority of NeuN^+^ cells are also positive for VGLUT1 (green) by DIV 21. However, many NeuN^+^ cells are positive for GAD2 (green) by DIV 21, with notable examples of GAD2- cells. Scale bars: DIV 7 (VGLUT1) 200 μm, all others are 50 μm.

**Figure 6:**
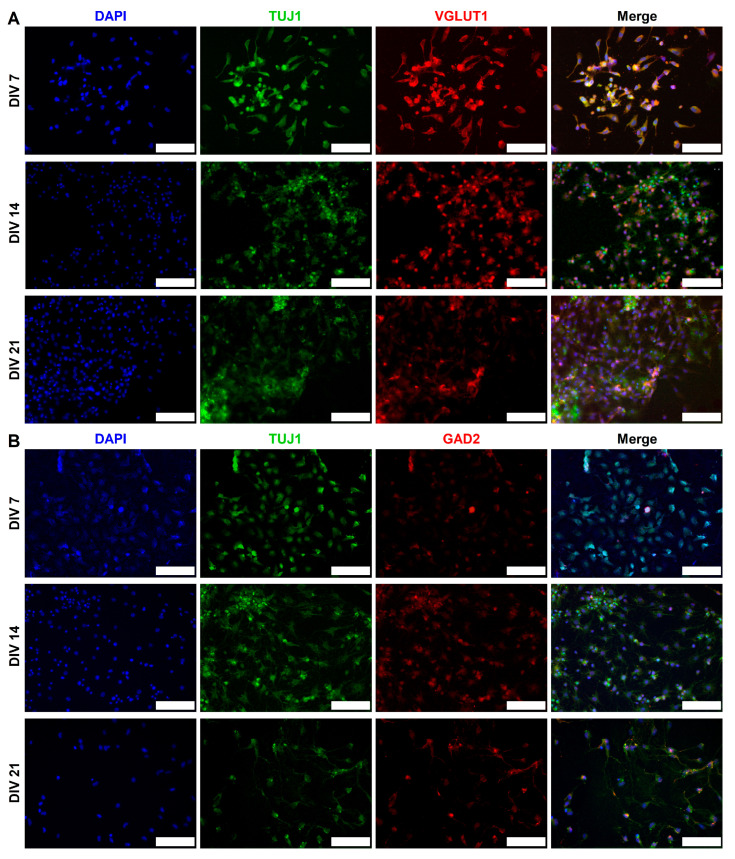
Examples of (**A**) VGLUT1 and (**B**) GAD2 expression in TUJ1^+^ (green) mESC-differentiated neurons at DIV 7, 14, and 21. VGLUT1 (red) expression in TUJ1^+^ cells remains stable across different time points. There is a noticeable increase in GAD2 (red) expression from DIV 7 to DIV 21. All scale bars: 50 μm.

**Figure 7:**
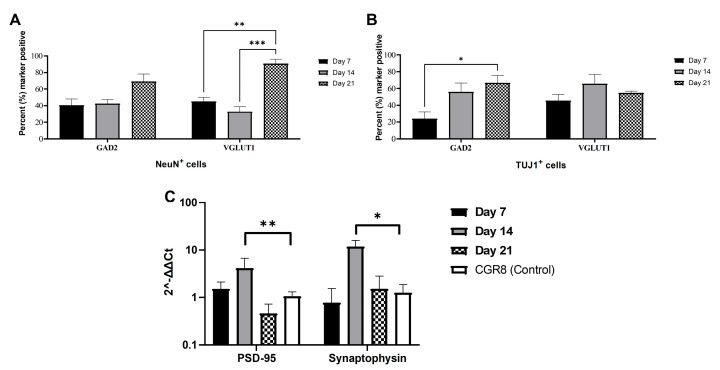
Quantification of the synaptic markers GAD2, VGLUT1, Synaptophysin, and PSD-95 in mESns. (**A**) Percentage of GAD2^+^ and VGLUT1^+^ neurons in NeuN^+^ cell populations at DIV 7, 14, and 21. (**B**) Percentage of GAD2^+^ and VGLUT1^+^ neurons in TUJ1^+^ cell populations at DIV 7, 14, and 21. (**C**) Gene expression of pre-synaptic (Synaptophysin) and post-synaptic (PSD-95) markers in mESns at DIV 7, 14, and 21. Undifferentiated CGR8 cells were used as a control. (All data are expressed as the means ± SEMs from three separate experiments, *n* = 6, * *p* < 0.05, ** *p* < 0.01, and *** *p* < 0.001).

CGR8 cell-derived neurons expressed excitatory (VGLUT1) and inhibitory (GAD2) synaptic markers. We monitored the expression of both of these markers in NeuN^+^ (Figure 5) and TUJ1^+^ (Figure 6) neurons across 3 weeks in culture. GAD2 expression was higher in NeuN^+^ neurons compared to TUJ1^+^ neurons, particularly at early stages of differentiation (DIV 7). The expression of VGLUT1 was similar across NeuN^+^ and TUJ1^+^ neurons, although we did observe a sharp increase in DIV 21 NeuN^+^ neurons. We measured GAD2 and VGLUT1 puncta and calculated the percentage of neurons that expressed each marker at DIV 7, 14, and 21 in NeuN^+^ (Figure 7A) and TUJ1^+^ (Figure 7B) neurons independently. GAD2 expression in NeuN^+^ neurons showed no statistically significant differences across DIV 7 (40.6% ± 7.4%, *n* = 6), DIV 14 (42.5% ± 4.8%, *n* = 6), and DIV 21 (69.6% ± 8.8%, *n* = 6). However, a two-way ANOVA comparing VGLUT1 expression (Tukey’s multiple comparison test) revealed a statistically significant increase in the number of VGLUT1^+^ neurons from DIV 7 (45.2% ± 5.0%, *n* = 6) and DIV 14 (32.9% ± 5.9%, *n* = 6) to DIV 21 (90.8% ± 5.3%, *n* = 6) (*p* = 0.0037 and *p* = 0.0002, respectively) (Figure 7A). Focusing on TUJ1^+^ neurons, there was a statistically significant increase in GAD2 expression from DIV 7 (24.0% ± 8.2%, *n* = 6) to DIV 21 (66.7% ± 8.8%, *n* = 6) (*p* = 0.0144) (DIV 14 (56.0% ± 10.5%, *n* = 6)) (Figure 7B). No statistically significant differences were found in VGLUT1 expression across DIV 7 (45.6% ± 7.3%, *n* = 6), DIV 14 (65.7% ± 11.2%, *n* = 6), and DIV 21 (54.7% ± 2.0%, *n* = 6) in TUJ1^+^ neurons.

Differentiated neurons exhibited a marked increase in the gene expression of the pre- and post-synaptic markers synaptophysin and PSD-95, which was mostly evident at DIV 14 (Figure 7C). Real time PCR values (2^−ΔΔCt^) for PSD-95 were 1.52 ± 0.6 at DIV 7, 4.15 ± 2.65 at DIV 14, and 0.46 ± 0.26 at DIV 21 and were contrasted to undifferentiated CGR8 cells (1.06 ± 0.24). Only the DIV 14 values for PSD-95 were significantly increased compared to the control CGR8 cells (*p* = 0.0099). Similarly, Synaptophysin values were 0.78 ± 0.78 at DIV 7, 11.84 ± 4.05 at DIV 14, and 1.54 ± 1.29 at DIV 21 and were contrasted to undifferentiated CGR8 cells (1.26 ± 0.61). Again, the DIV 14 values of Synaptophysin were significantly increased compared to the control CGR8 cells (*p* = 0.0265).

### 2.4. Functional Maturation of CGR8 Cell-Derived Neurons and Formation of Local Networks

**Figure 8:**
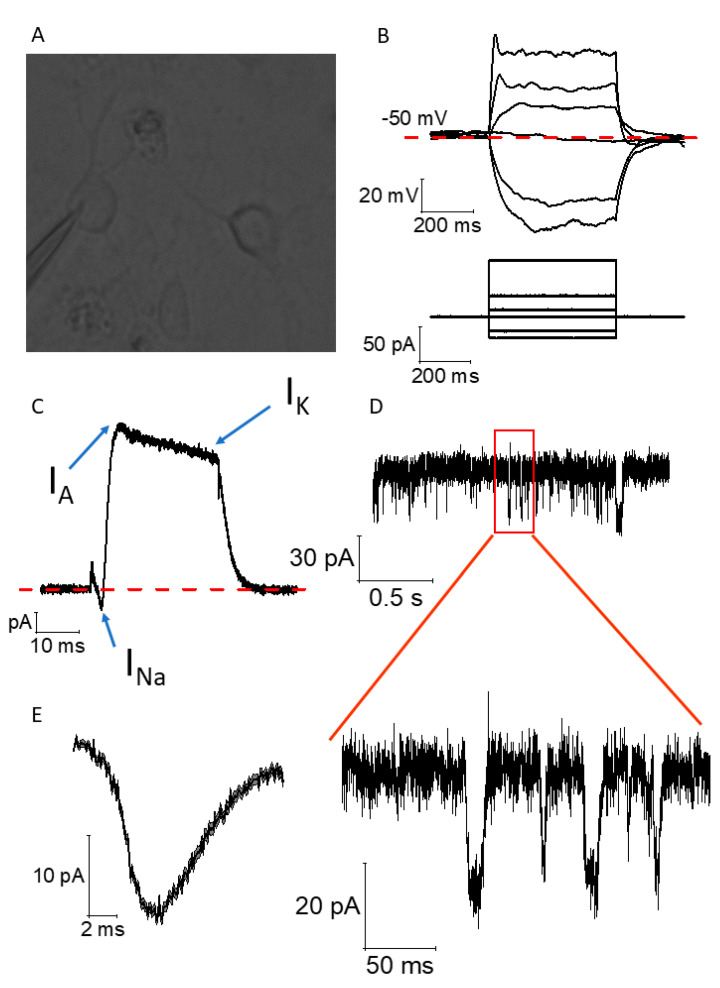
The differentiation protocol can induce functional mature single neurons and local networks. (**A**) Example of an FOV during a patch-clamp experiment. The pipette is attached in “whole-cell” mode. Some branching of the cell is visible. (**B**) Example traces of current-clamp recordings performed in CGR8 cells differentiated into neural-like cells. A square current, 500 ms, and negative and positive stimuli were applied to characterize the electrotonic and electrogenic properties of the cell. A “spikelet” is visible for a stimulation exceeding 20–25 pA. (**C**) The spikelet observed in B is underlined within the same cell by inward and outward currents evoked upon the depolarization of the cell from -90 mV to 0 mV for 30 ms in voltage-clamp configuration and following leak-subtraction. The recorded currents likely correspond to a weak inward I_Na_ followed by a fast-inactivating outward current (likely I_A_ mediated by fast inactivating voltage-gated potassium channels) and by a non-inactivating outward current (likely I_K_, mediated by non-inactivating, voltage-gated potassium channels). All recordings shown in (**B**,**C**) were conducted at room temperature. (**D**) Example of a voltage-clamp, gap-free recording conducted at a Vh = -70 mV in a CGR8 cell-derived neural-like cell. Both the top and bottom scales allow the visualization of single spontaneous excitatory post-synaptic currents, suggesting that the cultured cells form active neural networks. (**E**) Average ± SEM boundaries of the 140 events detected in the same cell. The recordings shown in (**D**,**E**) were carried out at 28 °C.

CGR8 neurons differentiated for 14–21 DIV showed neurophysiological phenotypes associated with partial functional maturation. Patch-clamp, whole-cell, current-clamp, and voltage-clamp recordings were carried out on neurons developing for 14 to 21 DIV (Figure 8A). Upon the injection of negative and positive square currents lasting 500 ms (Figure 8B, bottom panel), we observed negative and positive voltage deflections, respectively, following an exponential decay trend (negative) and a logarithmic increase (positive) typical of RC circuits, such as biological cell membranes. At currents exceeding 20–25 pA, we observed spikelets of voltage, often considered an immature form of an action potential (Figure 8B, top panel). Thanks to voltage-clamp whole-cell recordings, we also observed that these spikelets were associated, within the same cell, with short-lasting and tiny inward currents (presumably voltage-gated Na+ channel-dependent, I_Na_), fast inactivating (I_A_), and non-inactivating (I_K_) outward currents, presumably corresponding to the gating of fast-inactivating and non-inactivating voltage-gated K+ channels (Figure 8C). The presence of I_Na_ and I_A_ currents, and the relatively small amplitude of I_Na_, reinforces the idea of the partial maturity of these neurons. Finally, within the same example cell, we can observe the presence of spontaneous excitatory post-synaptic currents recorded at a holding voltage Vh = −70 mV using voltage-clamp recordings. In this example cell, we can observe multiple events (Figure 8D), which are summarized in the average ± SEM event shown in Figure 8E. This event exhibits the duration, decay, and recovery phase typical of an AMPA-R-mediated event.

## 3. Discussion

In the present study, we differentiated the mouse embryonic stem cell line CGR8 into neuronal cells that expressed mature neuronal markers and exhibited electrical activity. The majority of neuronal differentiation protocols are based on a two-step approach: the initial induction of stem cells into neural precursors and subsequent neuronal specification [36,37]. Two main morphogens driving neural induction are retinoic acid (RA) and smoothened agonist (or purmorphamine), which are typically applied immediately after embryoid body formation by the ESCs [36,37,38]. Withdrawal of LIF from ESC media in the absence of any growth factors leads to pluripotency loss, as stem cells start to “drift” toward a variety of lineages. Even though these are non-neuronal cells, they might exhibit neuronal attributes, such as an upregulated synthesis of acetylcholine [39]. Wichterle H. and Peljto M. established differentiation protocol based on varying concentrations of RA and smoothened agonist, producing motor neurons of varying rostro-caudal identities [32,40]. The combined use of retinoic acid and a smoothened agonist is efficient in neural induction, as these two morphogens activate complementary patterning pathways (Hox and Shh). Most such protocols require more than 2 weeks to generate neurons, which are characterized by the expression of markers such as Nestin, ISL1, TUJ1, and MAP2 [37,41,42]. Here, we commit the CGR8 cells to neural lineages in just 5 days and produce the mESns within a week later with the use of RA and purmorphamine, a low-cost, direct agonist of Smo that activates Hh signaling. Then, a neuronal media containing B27 and BDNF further enhances neuronal differentiation and growth.

During embryonic development, radial glia cells undergo asymmetric division into intermediate progenitor cells (IPCs). IPCs then symmetrically divide into two immature neurons (TUJ1^+^), which develop into different subtypes of neurons (e.g., glutamatergic neurons, GABAergic neurons, cholinergic neurons, etc.). Depending on their terminal identity, these mature neurons will express a variety of different markers (NeuN, Synaptophysin, MAP2, PSD95, etc.). The neurons derived in this study form interconnected clusters (Figure 1C), which is a typical cellular behavior of neuronal cultures. The derived cells also express both immature (TUJ1) and mature (NeuN) neuronal markers (Figure 2 and Figure 3). The expression of both neuronal markers (Figure 1A, TUJ1, NeuN) increases throughout the differentiation period (DIV 7 to 21), which is a clear indication of neuronal maturation [43]. Intriguingly, NeuN is expressed both in the nucleus and the cytoplasm of a few differentiating neurons. This has been documented in prior studies and linked to neuronal size and type (e.g., motor neurons) [44,45]. However, in this study, we did not assess markers of different neuronal subtypes (e.g., motor neurons, cholinergic neurons, etc.), as we make no claims on neuronal specification. Our proposed protocol simply produces active neurons that perhaps can be further driven into specific neural lineages. Oct4 expression sharply drops in DIV 7 differentiated neurons, which is a clear sign of pluripotency loss. The morphological data (Figure 1B) reinforce our hypothesis of ongoing neuronal maturation in the cultures, as the average number and length of neurites per cell increase from DIV 7 to 14 and stabilize at DIV 21, within the expected physiological range [46]. However, at DIV 14 and DIV 21, the average neurite length remains shorter than the respective average length in primary neuronal cultures. This has been observed in prior studies, with axonal growth being significantly shorter in stem cell-derived neuronal cultures compared to primary cultures [47]. It is easier to visualize neurites in sparse neuronal networks of early cultures (Figure 2, DIV7) than in the denser networks in DIV14–21 cultures (Figure 2, DIV 14 and 21). Therefore, in our calculation of neurite length, we measured both neurites and fascicles between clusters, when these were present in the collected images. Furthermore, we did not distinguish between axons and dendrites, as we used the TUJ1 marker to identify neurites in general. This could also explain the shorter average neurite length in our cultures.

The presence of both VGLUT1 and GAD2 (Figure 5 and Figure 6) signifies that RA and purmorphamine alone will not commit neurons exclusively to either an excitatory or inhibitory lineage. Despite the low levels of expression early in differentiation (DIV 7), at DIV 21, we observe a marked increase in both VGLUT1 (Figure 7A, NeuN+ neurons) and GAD2 (Figure 7B, TUJ1+ neurons) expression. The expression of synaptic markers (VGLUT1) has been used in other protocols as evidence of neuronal maturation [48]. Both excitatory and inhibitory synaptic markers are present in the mESC-derived neurons. The implication of our results in Figure 7A, B seems to be that DIV 21 NeuN^+^ neurons and DIV 14 and 21 TUJ1^+^ neurons co-express GAD2 and VGLUT1. However, our results are derived from different cell cultures stained for these different markers. We did not co-stain the same cell population for both VGLUT1 and GAD2. Therefore, statistically it is possible to have two average percentages of expression that add up to more than 100%, yet there is no co-expression of the two markers. This apparent contradiction arises from the way averages are calculated across multiple samples and fields of view, not from true cellular overlap.

Pre- and post-synaptic gene expression is upregulated (Synaptophysin and PSD-95, Figure 7C), which is a sign of functional maturation in neuronal networks. This is evident in the increase in the expression of these genes in DIV 14 cultures. There is a decrease at DIV 21; however, this can be explained by the potential presence of undifferentiated or partially differentiated cells that continue to proliferate. This also explains the relative increase in Oct4 expression in DIV 21 cells (Figure 4B). Cells that are either TUJ1^−^ (Figure 2) or NeuN^−^ (Figure 3) can be seen at later developmental points (DIV 14 and 21) in our cultures. Low purity and contamination of desired cell types with other cells is a common challenge found in many stem cell differentiation protocols [49].

We provide evidence of electrical activity in the network with the recordings of spontaneous post-synaptic currents (Figure 8D,E) and the presence of a spikelet upon current stimulation (20+ pA). Our functional data, while showing neurophysiological markers associated with partial maturity, such as spikelets and voltage-gated Na^+^ currents, do not allow us to claim full electrophysiological maturation of the mESns. We would expect electrical responses that are larger in amplitude from fully mature neurons. Similar phenotypes of stem cell-derived neurons have been observed by many research groups, which follow either short [50] or very long periods of time in culture [51]. This suggests that culture time is not the only factor responsible for functional maturation in neural networks. The evidence shows that the inclusion of different growth factors in the media can lead to better neuronal differentiation in a relatively short time [52], with the chemical composition of the medium being a key component that leads to mature action potentials and post-synaptic potentials [52]. Multiple studies have also identified astrocytes as critical partners in the initiation and shaping of neuronal activity [53,54,55]. Neuron–astrocyte entanglement is not limited to the cellular level but rather affects network activity, as astrocytes participate in synapse formation and elimination, thus dynamically remodeling the entire neuronal network [56]. In stem cell-derived neuronal cultures, the lack of astrocytes likely constrains neuronal maturation. Ultimately however, the epigenetic reset of stem cells is responsible for the difficulty in stem cell-derived neurons reaching full functional maturation, as this requires transcriptional processes that are gated by epigenetic changes in the host’s DNA at different stages of its development [57]. For this reason, it would be reasonable to introduce chemical cues that specifically promote functional maturation in our protocol.

What other factors might play a role in the maturation process? We had previously reported that mES cells and mESns at these time points express the classical nuclear hormone receptor ERα and the atypical ERs GPER1 and ERα-36. ERα-66 or the full-length classical ERα isoform, as well as Erβ, are present on cortical embryonic and adult rat NPCs, where they increase neuronal differentiation and decrease glial differentiation of embryonic but not adult NPCs in a p21- and EGF-dependent manner [58]. The differentiation of mES to motor neurons is also increased by the activation of Erα [59]. Our previous localization data using mES cells and mESns suggest that as neuronal differentiation proceeds, the redistribution of ERs occurs. Transcription in the mESn nucleus is most likely due to ERα, while non-genomic signaling initiated by the ERs from the plasma membrane likely increases in mESns due to the movement of all receptors out of the nucleus and into the plasma membrane [22]. Indeed, the repression of ERα in neuroblastoma cells decreased differentiation, while its replacement resulted in increased neurogenesis and redifferentiation [60]. It is possible that increased plasma localization during maturation of the classical ERα-66 as well as of GPER1 and ERα-36 allows for greater crosstalk with growth factor receptors, with concomitantly higher ERK signaling. ERK signaling and increased cAMP levels due to growth factor activation have been shown to increase neurite outgrowth in a neuronal differentiation model, i.e., the PC12 cell line [61] and in stem cells [62].

## 4. Materials and Methods

### 4.1. Cell Culture

The CGR8 mouse embryonic stem (mES) cell line was purchased from Sigma (Sigma & Aldrich, Gillingham, UK). The cells were maintained in an undifferentiated state in DMEM (Dulbecco’s Modified Eagle Medium) supplemented with 10% Fetal Calf Serum, 1% penicillin/streptomycin, 1% L-glutamine, and 100 µM 2-mercaptoethanol, along with 10^3^ units/mL of Leukemia Inhibitory Factor (LIF, Merck Life Science, Gillingham, UK). The mES cells were passaged every two days at a 1:8 split ratio. CGR8 cells were used up to passage 15 and then discarded. For differentiation, a mass suspension protocol based on the study by Peljto et al. was used [40]. On day 0, mES cells were seeded in non-tissue culture-treated Petri dishes at a higher density of 50,000 cells/mL and allowed to form embryoid bodies (EBs) in ADFNK media (ADMEM/F12–Neurobasal medium 1:1, 10% Knockout Serum Replacement, 1% penicillin/streptomycin, 1% L-glutamine, and 100 µM 2-mercaptoethanol) without LIF. Fresh media was supplemented on days 2 and 5, but in contrast to the Peljto protocol, we did not split the EBs. On day 2, 1 µM retinoic acid (RA) and 1 µM purmorphamine were added. Higher concentrations of RA can be toxic to embryonic stem cells, while lower concentrations may not be as effective at promoting neural differentiation [63]. With regard to the concentration of purmorphamine, we followed similar protocols (e.g., Peljto et al. [40]) that use SAG at 0.5 μΜ. However, as purmorphamine is significantly less potent, it is common practice to use a higher concentration of purmorphamine when substituting it for SAG in neural differentiation protocols to achieve a similar level of Shh pathway activation. On day 6, EBs were collected, washed with PBS, and dissociated with Accutase for 10 min at 37 °C, which breaks up the EBs and releases individual cells into the suspension. Accutase was neutralized with aggregation medium, and trituration was performed with three glass pipettes of varying bore sizes to increase the number of single cells. Cells were then centrifuged at 180× *g* for 5 min, and the supernatant was removed. The cells were resuspended in aggregation medium, passed through a 70 µm cell strainer to eliminate large aggregates and matrix, and the cell density was counted using a hemocytometer. Individual cells were plated at a density of 150–200 cells/mm^2^ on laminin-coated glass coverslips (2 µg/cm^2^) and incubated in ADFNB media (ADMEM/F12–Neurobasal medium 1:1, 1x B-27 supplement, 1% penicillin/streptomycin, 1% L-glutamine, 5 ng/mL BDNF, and 100 µM 2-mercaptoethanol) at 37 °C, 5% CO_2_ for 7–21 days in vitro (DIV). The data presented in this study were derived from three independent replicate experiments.

### 4.2. Immunohistochemistry

Differentiated neurons (mESns) on coverslips were fixed with 4% paraformaldehyde (PFA) for 20 min at room temperature (RT) at 7, 14, and 21 days in vitro (DIV) to assess neuronal maturation. The cells were washed three times with PBS for 5 min each (Thermofisher, Birmingham, UK) and then permeabilized with a solution containing 10% normal goat serum (NGS) (Fisher, UK) and 0.1% Triton X-100 (Sigma, Gillingham, UK) in PBS for 20 min on a shaker at RT in 24-well plates. After permeabilization, the cells were blocked with SuperBlock (Thermofisher, Birmingham, UK) and 0.5% Triton X-100 for 1 h at RT. A primary antibody incubation solution was prepared by mixing 10% NGS and 0.5% Triton X-100 in SuperBlock, along with the primary antibodies listed in Table 1. The cells were incubated with the primary antibodies overnight on a shaker at 4 °C. Afterward, the coverslips were washed four times with PBS for 10 min each time at RT before an incubation with the secondary antibodies (see Table 1). If a second or third antigen was to be detected in the same cells, the immunocytochemistry (ICC) process was repeated. After a final round of PBS washes (four times, 5 min each at RT), the coverslips were mounted using Fluoromount-G Mounting Medium with DAPI and sealed with DPX mounting medium. The mounts were allowed to cure for 24 h at RT in the dark, followed by storage at 4 °C.

### 4.3. Image Analysis

Cells on coverslips were imaged using a Zeiss AxioImager microscope with 5×, 10×, and 20× objective lenses (Zenn 3.4 Blue software). Images obtained with the 20× objective were used for quantification. Negative control images were used to determine the exposure threshold for each fluorophore, and all samples were then imaged below this threshold. For each experimental time point, a minimum of two coverslips were fixed and imaged. Using the 20× objective, up to four images were captured across the coverslips for analysis. Images were analyzed with ImageJ 1.54n by split mining the three channels and identifying neurons based on β-tubulin-III (TUJ1) or NeuN staining. A random selection of 25–30 neurons from each coverslip was marked using the region of interest (ROI) tool. The ROI was then overlaid with the other channels to count VLGUT and GAD2 puncta in neurons. The percentage of VLGUT-positive and GAD2-positive neurons was calculated, and the data were graphed using GraphPad Prism 8.

For the visualization and quantification of Oct4 expression, we collected images from three different coverslips using a 10× objective for a broader field of view. Images were analyzed with ImageJ 1.54n, and the total pixels for each channel (marker and nuclei-DAPI) were measured. Obvious artifacts that would skew the fluorescence data were excluded from the analysis. The ratio of marker to nuclei pixels was calculated and graphed using GraphPad Prism 8.

To measure neuronal dendrite length (or protrusions) and soma diameter, the freehand tool in Fiji was used. Twenty-five (25) β-tubulin-III (TUJ1)-positive or NeuN-positive neurons were selected from each 20× image across all replicate experiments and time points. Data were analyzed using GraphPad Prism 8 to create plots showing the means ± SEMs. One-way ANOVA was performed to assess group differences, with a *p*-value of <0.05 considered statistically significant.

### 4.4. RNA Extraction and cDNA Synthesis

Neurons at 7, 14, and 21 days in vitro (DIV) were cultured at a density of 160 cells/mm^2^ in 6-well laminin-coated plates (2 μg/cm^2^). At 70–80% confluence, cells were trypsinized, centrifuged to form a pellet, and resuspended in RNA Later. The samples were stored at −20 °C. RNA extraction was carried out with the RNAeasy kit (Qiagen Inc. Manchester, UK). RNA samples with a 260/280 ratio between 1.8 and 2.1 were selected for cDNA synthesis. cDNA (5 ng/µL) from 7, 14, and 21 DIV neurons was generated from reverse transcribed total RNA (2 µL of 10× reverse transcriptase (RT) buffer, 0.8 µL of 25× 10 mM dNTPs, 2 µL of RT random primers, and 1 µL of multi-scribe RT), using the High-Capacity cDNA Reverse Transcription Kit (Thermofisher, Birmingham, UK). The reaction conditions included an initial incubation at 25 °C for 10 min, followed by 37 °C for 120 min and a final step at 85 °C for 5 min.

### 4.5. Real-Time PCR

For the time points 7, 14 and 21 DIV, 1 µL of cDNA (5 ng/µL), 7 µL of 2x SYBR Green mix (Fisher, Birmingham, UK), 1 µL of forward primer (5 µM stock), and 1 µL of reverse primer (5 µM stock) (IDT, Leuven, Belgium) were used in a 96-well plate to amplify target genes (synaptophysin, PSD-95, and β-actin as the housekeeping gene) in duplicate. The PCR cycling conditions were as follows: initial hold at 95 °C for 15 min, followed by 40 cycles of denaturation at 94 °C for 15 s, annealing at 60 °C for 30 s, and extension at 72 °C for 30 s. CGR8 murine stem cells were included as a control to compare gene expression between neurons and CGR8 cells. The ∆∆Ct value was calculated by subtracting the ∆Ct of the reference sample average from the ∆Ct of the sample. The 2^−∆∆Ct^ value was then computed and plotted with SEMs using GraphPad 8.

### 4.6. Electrophysiology

Patch-clamp recordings of GCR8 neurons grown on laminin-coated glass coverslips were performed after 14–21 DIV. The cells were submerged in Hank’s Balanced Salt Solution (HBSS) containing 138 mM NaCl, 5 mM KCl, 0.4 mM KH_2_PO_4_, 0.3 mM Na_2_HPO_4_ anhydrous, 4.2 mM NaHCO_3_, 0.5 mM MgCl_2_, 0.4 MgSO_4_, 1.3 mM CaCl_2_, and 5.5 mM glucose, pH 7.3, at room temperature of 25 °C and 290–300 mOsm. Cells were visualized using an inverted microscope using phase contrast (Olympus, Tokyo, Japan). Standard glass micropipettes with a resistance of 2.5–6 MΩ were used for recordings, and filled with an internal solution containing 145 mM K-gluconate, 5 mM NaCl, 10 mM HEPES free acid, 0.2 mM EGTA, 0.3 mM Na-GTP, and 4 mM Mg-ATP, pH 7.3, 280–290 mOsm. The liquid junction potential (JPC) that arose from the combination of bath and pipette solution was corrected arithmetically.

All measurements were made using a Multiclamp 700B patch-clamp amplifier (Molecular Devices, San Jose CA, USA). Data were low-pass filtered (5–10 kHz), digitized (20–100 kHz), and subsequently visualized and stored on a PC using pClamp 10 electrophysiology software. Following entry into the whole-cell recording configuration, pipette capacitance was neutralized and the series resistance was compensated (10–80% correction). Voltage-clamp (VC) recordings were made for the quantitative evaluation of voltage-gated currents; starting from a holding voltage of −90 mV, 12 voltage steps (10 mV for 30 ms) were applied and the consequent voltage-gated currents were measured. After VC recordings were completed, the amplifier was switched to I = 0 current injection and the resting membrane potential (RMP) was assessed. Subsequent current-clamp (CC) recordings were performed to assess passive properties and eventual action potential generation; they consisted of the application of 1515 square current pulses of 500 ms (−30, −20, −10, 0, 10, 20, 30, 40, 50, 60, 70, 80, 90, 100, and 110 pA); these recordings were performed whilst keeping the pre-stimulus membrane voltage Vm at −80 mV with a constant current injection to avoid biases arising from cell-to-cell variability in the RMP. Finally, sEPSCs were recorded in voltage-clamp mode at a holding potential V_h_ = −70 mV, running a gap-free protocol for about 60 s. The sEPSCs were identified using a template search function embedded into Clampfit 10.7.

## 5. Conclusions

In summary, we derived functional neurons from mESCs in under two weeks. Through an embryoid body induction protocol followed by a 3 week maturation period, cells with diverse expression profiles of TUJ1, NeuN, GAD2, and VGLUT1 emerged. This is a heterogeneous neuronal population at different stages of development. The differentiated neurons have a soma of 30 μm in diameter and develop on average 4 neurites, with their longest neurite being 70 μm in length. The derived neurons also exhibit “spikelets” for stimulations exceeding 20 pA. Single whole-cell recordings highlight the presence of inward sodium currents, followed by outward currents, which are likely caused by fast-inactivating, voltage-gated potassium channels. We also demonstrate the upregulation of genes encoding the pre- and post-synaptic markers Synaptophysin and PSD-95 during the second week of culture (DIV 14), suggesting that differentiated neurons can form networks with mature synapses. The simplicity, reproducibility, and speed of our protocol facilitate its use in multiple future investigations.

## Figures and Tables

**Table 1:** Primary and secondary antibodies used and their dilutions.

Target	Company/Cat No.	Host	Dilution
Β-Tubulin III (TUJ1)	Abcam, Cambridge, UK (ab 18207)	Mouse	1:300
VGLUT1	2BScientific Kidlington, UK (135-316-SY)	Chicken	1:300
GAD2	St Johns (STJ23739)	Rabbit	1:500
Rabbit	ThermoFisher, UK (A-11011)	Goat	1:300
Chicken	ThermoFisher, UK (A-21449)	Goat	1:300
Mouse	ThermoFisher, UK (A-11001)	Goat	1:300

## Data Availability

The data underlying this article will be shared upon reasonable request to the corresponding author.

## Abbreviations

The following abbreviations are used in this manuscript:

LIF	Leukemia Inhibitory Factor
mESC	Mouse embryonic stem cell
RA	Retinoic acid
RMP	Resting membrane potential
